# The Role of Intestinal Dysbacteriosis Induced Arachidonic Acid Metabolism Disorder in Inflammaging in Atherosclerosis

**DOI:** 10.3389/fcimb.2021.618265

**Published:** 2021-03-18

**Authors:** Yingxin Sun, Danbin Wu, Wenyun Zeng, Yefei Chen, Maojuan Guo, Bin Lu, Huhu Li, Chun Sun, Lin Yang, Xijuan Jiang, Qing Gao

**Affiliations:** ^1^ School of Integrative Medicine, Tianjin University of Traditional Chinese Medicine, Tianjin, China; ^2^ Department of Rheumatology, The First Affiliated Hospital of Guangzhou University of Chinese Medicine, Guangzhou, China; ^3^ First Clinical Medical School, Guangzhou University of Chinese Medicine, Guangzhou, China; ^4^ Guangdong Key Laboratory for Biomedical Measurements and Ultrasound Imaging, School of Biomedical Engineering, Shenzhen University Health Science Center, Shenzhen, China

**Keywords:** atherosclerosis, aging, inflammaging, intestinal flora, serum metabolomics

## Abstract

**Background:**

Aging induced chronic systemic inflammatory response is an important risk factor for atherosclerosis (AS) development; however, the detailed mechanism is yet to be elucidated.

**Objective:**

To explore the underlying mechanism of how aging aggravates AS advancement.

**Methods:**

A young (five-week-old, YM) and aged group (32-week-old, OM) male apoE^-/-^ mice with a high fat diet were used as models, and age-matched male wild-type C57BL/6J (WT) mice were used as controls. AS lesion size, serum lipid profile, cytokines, and gut microbiota-derived LPS were analyzed after 32 weeks of diet intervention. A correlation analysis between the 16S rRNA sequencing of the feces and serum metabolomics profiles was applied to examine the effect of their interactions on AS.

**Results:**

ApoE^-/-^ mice developed severe atherosclerosis and inflammation in the aorta compared to the WT groups, and aged apoE^-/-^ mice suffered from a more severe AS lesion than their younger counterparts and had low-grade systemic inflammation. Furthermore, increased levels of serum LPS, decreased levels of SCFAs production, as well as dysfunction of the ileal mucosal barrier were detected in aged mice compared with their younger counterparts. There were significant differences in the intestinal flora composition among the four groups, and harmful bacteria such as *Lachnospiraceae_FCS020*, *Ruminococcaceae_UCG-009*, *Acetatifactor*, *Lachnoclostridium* and *Lactobacillus_gasseri* were significantly increased in the aged apoE^-/-^ mice compared with the other groups. Concurrently, metabolomics profiling revealed that components involved in the arachidonic acid (AA) metabolic pathway such as 20-HETE, PGF2α, arachidonic acid, and LTB4 were significantly higher in the aged AS group than in the other groups. This suggested that metabolic abnormalities and disorders of intestinal flora occurred in AS mice.

**Conclusions:**

Aging not only altered the gut microbiome community but also substantially disturbed metabolic conditions. Our results confirm that AA metabolism is associated with the imbalance of the intestinal flora in the AS lesions of aged mice. These findings may offer new insights regarding the role of gut flora disorders and its consequent metabolite changed in inflammaging during AS development.

## Introduction

Aging is an independent risk factor of developing atherosclerosis, which generally involves medium and large arteries and is characterized by the formation of a fibrous plaque or atheroma in the arterial intima. This pathological process initiates from damaged endothelium that promotes aggregation of cholesterol-containing LDL particles which were oxidized at the arterial wall, then inflammatory responses ensue (Tsilingiri et al., 2019). It was shown that even in the absence of an atherosclerosis vessel, aging per se can lead to intimal and medial thickening and vascular stiffness, which were also seen in AS (North and Sinclair, 2012). Furthermore, chronic inflammation is a very important factor that promotes the progression of AS (Bäck et al., 2019). Aging is associated with the increased expression of adhesion molecules and proinflammatory cytokines that further contribute to the inflammation process (Libby et al., 2018). As the hallmark of AS, inflammation facilitates the fissure, erosion, and rupture of the aortic plaque (Zhong et al., 2020). Most importantly, targeting inflammation is applied as the essential therapy to treat AS in clinic (Lawler et al., 2020).

Advanced age is associated with immune dysregulation, which increases the risk of developing cardiovascular diseases (Ras et al., 2013). Aged individuals are liable to developing a pro-inflammation status characterized by elevated levels of pro-inflammatory markers without evident triggers. This condition was named as inflammaging by Claudio Franceschi in 2000 (Franceschi, 2000). It was then revealed that inflammaging aggravates vascular pathology and drives AS (Ferrucci and Fabbri, 2018). Possible mechanisms of inflammaging include cellular senescence, genetic predisposition to diseases, NLRP3 inflammasome activation, oxidative stress, central obesity, increased gut permeability and changes of microbiota composition subjective to lifestyle and diet (Grebe et al., 2018; Brandsma et al., 2019).

With the increase of life expectancy, understanding the effects of inflammaging on AS would benefit public health (Thum and Sedding, 2020). Aging can be identified by a number of senescence-associated biomarkers. Under circumstances with gut microbiota dysfunction, the homeostasis between bacteria and the host is disrupted, resulting in the accumulation of endotoxin but the reduction in short-chain fatty acids (SCFAs) (Tang et al., 2017). Both processes play crucial roles in inflammaging (Lindskog Jonsson et al., 2017). It was reported that aged intestinal flora contributes to systemic inflammaging by being transferred to young germ-free mice (Fransen et al., 2017). Apolipoprotein E-deficient (apoE^-/-^) mice (C57 genetic background) are considered to be the ideal animal model for human lipoprotein disorders and AS studies (Meir and Leitersdorf, 2004). ApoE knockout, which has been created by homologous recombination in embryonic stem (ES) cells, always combines with a high-fat diet to exacerbate AS susceptibility (Plump et al., 1992). Although there are many studies evaluating the pathogenesis of AS in the apoE^-/-^ mouse model, few studies related to aging as an aggravating inflammatory response in AS development have been reported.

In this study, we hypothesize that gut flora modifications and its subsequent metabolite changes might potentially be associated with inflammaging during the advancement of AS. We then pathologically analyzed the AS lesion, assessed the serum lipid profile, and levels of inflammatory cytokines to examine how aging exacerbates advanced atherosclerotic development. Finally, LPS, ileal mucosal barrier function, SCFA production in feces, as well as the gut microbiota profile and serum metabolomics were further investigated and compared between WT mice and apoE^-/-^ mice, as well as between two different ages.

## Materials and Methods

### Animals and Experimental Procedures

Young (five-week-old) and aged (thirty-two-week-old) male apolipoprotein E-deficient (apoE^-/-^, n=10) mice and age-matched male wild-type C57BL/6J (WT, n=10) mice were purchased from HFK Bioscience (Beijing, China). After 1 week of acclimatization, young and aged apoE^-/-^ mice were fed a high fat diet (HFD: fat 21% and cholesterol 0.15%, MD12015, Medicience Ltd, Jiangsu) for 32 weeks to establish the young (group YM; 37 weeks) AS model group and aged AS model groups (group OM; 64weeks), respectively. Young and aged C57BL/6J mice were fed a normal chow diet for 32 weeks to be used as the young control group (group YC; 37 weeks) and aged control group (group OC; 64weeks), respectively. All mice were housed in a controlled environment (temperature of 22 ± 2°C and a 12 h day/night cycle) and were allowed to access tap water and standard mouse chow *ad libitum*.

### Sample Collection

Fecal samples were collected immediately after the period of diet intervention. Blood samples were then harvested through the angular vein after overnight fasting under anesthesia using ether inhalation. Blood was allowed to settle on ice for 30 min and was centrifuged at 3,000 r.p.m. for 15 min at 4°C for serum collection. The hearts and entire aortas were perfused with ice-cold phosphate-buffered saline. All the samples were stored at -80°C until further assessment.

### High Frequency Ultrasound Technology, Lipid and LPS Analysis

Mice were anesthetized by inhalation of ether, and a High-Frequency Ultrasound Imaging System (Vevo 2100, USA) and 40 MHz transducer were used to assess the plaque in the AS artery arch as previously reported (Clouet, et al., 2016).

Serum levels of total serum cholesterol (TC), triglyceride (TG), low-density lipoprotein cholesterol (LDL-C), and high-density lipoprotein cholesterol (HDL-C) were determined using commercially available kits (Nanjing Jiancheng Bioengineering Institute, Nanjing, China). Serum LPS concentrations were measured with the Elisa kit (CEB526Ge, Uscn Life Science Inc. Wuhan, China) according to the manufacturer’s instructions.

### Oil Red O and HE Staining

The entire aortas (from the aortic arch to the iliac bifurcation with brachiocephalic trunk) were isolated and split longitudinally, then fixed in 4% paraformaldehyde for 24 h. The fresh aortic root was then frozen in optimum cutting temperature (OCT) compound and sectioned to a thickness of 10μm. Furthermore, aortic root and terminal ileum tissues were fixed with 10% buffered formalin then imbedded in 10% paraffin before being sectioned to a thickness of 5μm. Samples were stained with Oil red O (0.5% in 60% isopropyl alcohol) for 10 min. The excess stain was removed by incubating in 60% of isopropyl alcohol for 2 min then store in PBS. The images were observed under a light microscope (Leica DM3000, Germany) and analyzed by Image Pro Plus software. The lesion area of the atherosclerotic plaque was calculated as the percentage of the total area.

### Luminex Based Multiplex Cytokine Detection

Serum cytokines including granulocyte-monocyte colony-stimulating factor (GM-CSF), monocyte chemoattractant protein-1 (MCP-1), LPS-induced CxC chemokine (LIX), soluble CD40 ligand (sCD40L), INF-γ, interleukin (IL)-1β, IL-6, IL-7, IL-8, IL-10, IL-12, IL-17, and TNF-α, were simultaneously measured using mouse cytokine multiplex kits (MHSTCMAG-70K, Millipore, Billerica, MA, USA), according to the manufacturer’s instructions. The data were acquired using Luminex xPONENT^®^ software (v.3.1) and the median cytokine/chemokine fluorescence intensity was acquired using the MasterPlex^®^ QT software (v1.1).

### Immunohistochemistry

Frozen aortic root sections adjacent to the sites of the maximum lesion area were stained with primary antibodies against CD68 (marker for macrophages, rabbit polyclonal; Abcam, catalog number ab125212), and terminal ileum tissues were stained with antibodies of zonula occludens-protein 1 (ZO-1, 33-1500, Thermo Fisher Scientific), Occludin (OCLN, 339188, Thermo Fisher Scientific), toll like receptor 4 (TLR-4, sc-293072, Santa Cruz), and toll like receptor 9 (TLR9, sc-47723, Santa Cruz) respectively, followed by their corresponding secondary antibody conjugated to horseradish peroxidase (HRP, SV0002 or SV0001, BOSTER, Wuhan, China). Staining was illuminated by Diaminobenzidine (DAB), and pictures were taken by a Leica scanning electron microscope and analyzed using Image J software.

### Quantitative Real-Time PCR

Total RNA and protein were extracted from the entire aorta using the total DNA/RNA/Protein Kit (Invitrogen, CA, USA) according to the manufacturer’s instructions. Subsequently, the isolated mRNA was reverse transcribed into cDNA under the instruction of superscript reverse transcriptase (KR105-02, Tiangen Biotech, Beijing, China). The cDNA library was amplified with Taq DNA polymerase (Tiangen Biotech, Beijing, China). The sequences of primers were synthesized by Sangon Biotech (China) and are shown in **Table S1**. PCR reaction was completed after 40 cycles of 95°C for 30 s, 60°C for 1 min, and 72°C for 1 min. Relative fold-changes of gene transcriptions were calculated using the 2^−ΔΔCt^ method.

### Western Blotting

Protein quantification was conducted using a modified bicinchoninic acid assay (Cwbio, China). Protein samples were boiled for 5 min in loading buffer (Cwbio, China), separated by sodium dodecyl sulfate polyacrylamide gel electrophoresis on 12% gel, and then transferred onto the polyvinylidene fluoride membrane *via* electroblotting. After being blocked using skimmed milk for 1 h, the membranes were incubated at 4°C overnight with the primary antibodies, including ICAM-1 (ab179707, Abcam), VCAM-1 (ab134047, Abcam) and GAPDH (WL03413-020, Wanleibio, China), followed by incubation with the goat anti-rabbit horseradish peroxidase-conjugated secondary antibody (ab6721, Abcam) at room temperature for 1h. Protein bands were illuminated using an enhanced chemiluminescence kit (Cwbio, China) and visualized under a ChemiDoc imaging system (Bio-Rad Laboratories, Berkeley, CA, USA).

### 16S rRNA Gene Sequencing Analysis

Fresh mouse fecal samples were collected in separate sterile EP tubes immediately, after diet intervention. Total genomic DNA from the frozen fecal samples of mice was extracted using the hexadecyl trimethyl ammonium bromide method and were stored at -20°C until analysis. The V3 and V4 region of the bacterial 16S rRNA gene were amplified using specific primer (515F:5’-GTGCCAGCMGCCGCGGTAA-3’; 806R: 5’-GGACTACHVGGGTWTCTAAT-3’) in Phusion High-Fidelity PCR Master Mix (New England Biolabs). The TruSeq DNA PCR-Free Sample Preparation Kit (Illumina, USA) was used to generate Sequencing library. The DNA library was then sequenced using an Illumina HiSeq instrument at Novogene Bioinformatics Technology Corporation. Decultiplex of Raw Illumina fastq files were quality-filtered and analyzed using Quantitative Insights into Microbial Ecology (QIIME) version 1.7.0 (Caporaso et al., 2010). Sequence analysis was performed by Uparse v7.0.1001 (Edgar, 2013) (http://drive5.com/uparse/) and operational taxonomic units (OTUs) were clustered with a 97% similarity cutoff. A representative sequence for each OTU was screened for species annotation by Mothur methods and SSUrRNA databases (Quast et al., 2013) of SILVA(http://www.arb-silva.de/) (Wang et al., 2007) and OTU abundances were normalized using the standard sequence number corresponding to the sample with the least sequences.

### Short-Chain Fatty Acid Analysis by HS-GC/MS

The short-chain fatty acid (SCFA) analysis was used by a 7890A gas chromatography system (Agilent Technologies, Germany) coupled to a 5975c inert MSD quadrupole mass spectrometer, a 7697A headspace automatic injector, and a DB-FFAP capillary column (30 mm × 0.25 mm i.d., 0.25 μm film thickness). A portion of the 0.1 g homogenized fecal sample was mixed in 1 mL of 6% H_3_PO_4_ solution by ultrasound for 3 min and was then placed in an automatic headspace sampler at 80°C for 30 min. Subsequently, the samples (1 ml) were injected into the column at 80°C. Helium gas was used as a carrier for samples to pass through the column at a constant flow rate of 1 mL/min. The oven was preheated at 50°C for 1 min; then heated up to 200°C at the rate of 10°C/min. The temperature of the ion source and injector was set to 250°C. The mass detector system was operated in an electron impact (EI) mode with ionization energy of 70 eV. The data of ions monitored were collected from m/z 33 to 200. The short-chain fatty acids were quantified by the MS library of the National Institute of Standards and Technology (NIST 11).

### Serum Metabolomics

The serum samples were diluted with 300 μl chromatographic acetonitrile and mixed by ultrasound for 10 min followed by vortex. The mixture was then centrifuged at 13,000 rpm at 4°C, for 10 min to remove any sediment. Samples (5 μL) were analyzed by the UPLC-Q/TOF-MS system. The metabolites were separated by the waters Acquity UPLC I CLASS system (Waters, Milford, MA) with a HSS T3 column (2.1 mm × 100 mm, 1.7 μm, Waters U.K.), with the temperature set to 45°C. Solvent A (acetonitrile modified by the addition of 0.1% formic acid) and solvent B (0.1% formic acid in water) were used as the gradient elution. The experiment procedure and data acquisition for analysis were in consistence with the previous studies of Lili Song (Song et al., 2017).

### Bioinformatics Analysis

The alpha-diversity included Shannon, Chao1, Simpson, and ACE indices and was calculated using Mothur software version 1.30. The beta diversity was evaluated using QIIME, while Principal Component Analysis (PCA) was used to evaluate differences of samples in species complexity. Linear discriminant analysis (LDA) effect size (LEfSe) analyses (LDA score >4) were performed based on the output normalized data. LEfSe was used to identify taxa that were specific among each group.

Metabolomics statistical analyses were summarized as follows: the raw data of WT and apoE^-/-^ groups were collected by MarkerLynx Version 4.1 (Waters) based on UPLC-Q/TOF-MS. The raw data was processed for peak area calculation and peak normalization then filtered to identify potential discriminant variables. The exported data were processed using multivariate data analysis by SIMCA-P+ 14.0 software (Umetrics AB, Umea, Sweden). OPLS-DA was used in data processing of metabolomics, then the obtained score plot was used to establish a model visualization. Under circumstances of combining VIP >1(Variables with significant differences in their group contribution)and T <0.05 (T test for filtered data), molecular structures would be further identified.

### Statistical Analyses

Statistics were analyzed using SPSS 18.0, unless they were differently noted in the methods. Parametric variables were designated as mean ± SD and nonparametric variables were expressed as median with an interquartile range. Significant differences were calculated using the Student’s t test and nonparametric Mann-Whitney U test. *P <*0.05 was denoted as statistically significant.

## Results

### Atherosclerotic Lesions Analysis

**Figure 1:**
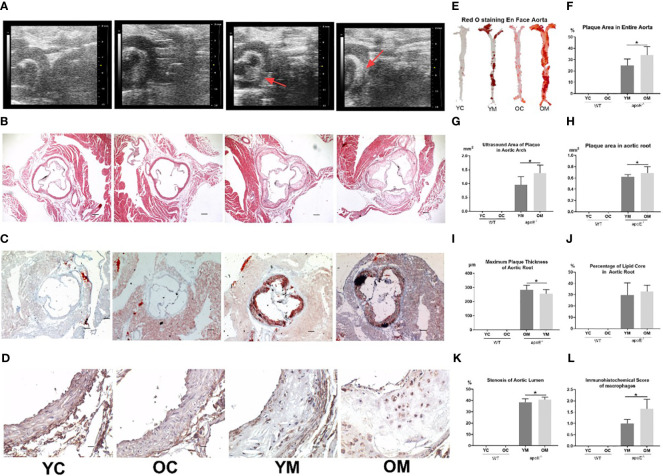
Quantification of AS, n=6 mice per group. **(A)** Representative images of aortic arch ultrasound, **(B)** HE and **(C)** Red O staining of aortic root sections (All images were taken at 40× magnification), and **(D)** immunohistochemical staining for macrophages infiltration in the atherosclerotic plaque of aortic root sections (All images were taken at 200× magnification); **(E)** En face analysis of aortas. Atherosclerotic lesions were identified by oil-red-O staining; **(F)** Total area of atherosclerotic lesion in the entire aortas; **(G)** Atherosclerotic plaque area in aortic arch (ultrasound); **(H)** Atherosclerotic plaque area in aortic root; **(I)**Thickness of maximum plague in aortic root; **(J)** The percentage of lipid core in atherosclerotic plaque of aortic root. **(K)** Percentage of stenosis at aorta lumen; **(L)** Score of macrophages infiltration in the atherosclerotic plaque in aortic root. Data were shown as mean ± SD. *p <0.05; *p* values are calculated from T-test.

Compared with the corresponding control groups, the atherosclerotic plaque areas of the aortic arch by high frequency ultrasound technology were increased in young (0.96 ± 0.29 mm^2^; *P*<0.01, **Figure 1A**) and aged (1.38 ± 0.29 mm^2^; *P*<0.01, **Figure 1G**) apoE^-/-^ mice with HFD for 32 weeks. The plaque area in the aortic arch of the OM group was significantly larger than that in the YM group (1.38 ± 0.29 mm^2^ *vs* 0.96 ± 0.29 mm^2^, *P*<0.05). Furthermore, the percentages of the plaque area in the entire aorta by Oil red O staining were also elevated in the OM group (33.99 ± 7.52%; *P*<0.01, **Figure 1E**) and YM group (24.83 ± 5.87%; *P*<0.01, **Figure 1F**) compared to the control groups. HE staining of the aorta root showed that the AS plaque area (0.69 ± 0.11 mm^2^ *vs*. 0.62 ± 0.04mm^2^, *P*<0.05), the stenosis of the aorta lumen (38.53 ± 3.13 *vs*. 40.74 ± 2.19%), and the maximum plaque thickness (282.44 ± 33.17 *vs*. 256.05 ± 29.34μm, *P*<0.05) were all much more severe in the OM group than those in YM (**Figures 1B, H, I, K**). However, Oil red O staining in the aortic root (**Figure 1C**) showed no significant difference in the percentage of the lipid core between the YM and OM group (**Figure 1J**).

### Lipid Profile and Serum Inflammatory Markers

Compared with their age matched WT groups, both YM and OM groups exhibited significantly higher serum TC (YM: 14.0-fold; OM: 12.4-fold, *P*<0.01), TG (YM: 7.6-fold; OM: 2.3-fold, *P*<0.01) and LDL-C (YM: 43.3-fold; OM: 39.3-fold, *P*<0.01) but significantly lower serum HDL-C (YM:0.8-fold, *P*<0.05) (**Supplementary Figure S1A–D**). Aging per se caused significant reduction of serum HDL-C as YC and OC was compared (*P*<0.05) or YM and OM were compared (*P*<0.01), as shown in **Supplementary Figure S1D**. Serum TC is significantly higher in OM compared with that in YM (*P*<0.05) while serum TG is significantly increased in the OC group than that in the YC group (*P*<0.05). No significant difference in serum LDL-C was observed between the YC and OC groups (**Supplementary Figures S1A, C**).

**Figure 2:**
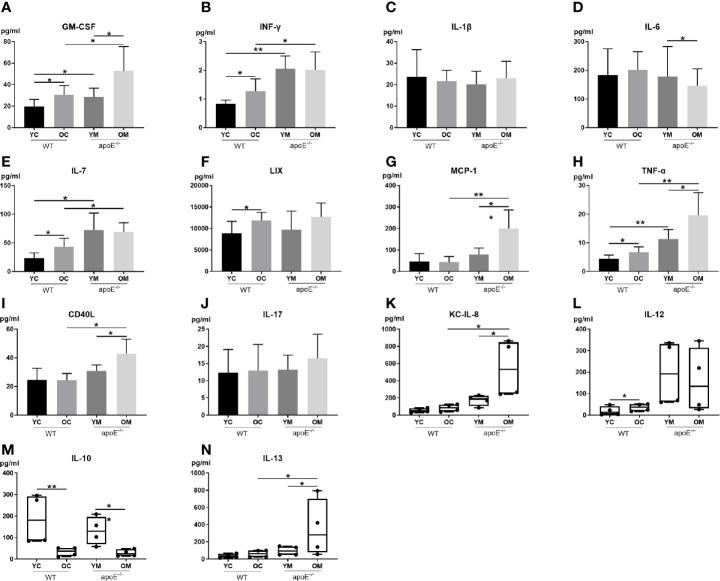
Quantification of serum inflammatory cytokines: **(A–J)** Data shown as mean ± SD; **(K-N)** Data were shown in boxplot format (horizontal bar represents the median, boxes encompass the 25^th^ to 75^th^ percentile and error bars encompass the 10^th^ to 90^th^ percentile). Data were shown as mean ± SD. ^*^ *p <*0.05; ^**^ *p <*0.01. *p* values are calculated from T-test and Wilcoxon rank sum test, n=6.

To explore the underlying mechanism of aging in aggravating plaque development, 14 serum inflammatory cytokines were examined (**Figure 2**). Serum levels of pro-inflammatory cytokines include GM-CSF, IFN-γ, IL-7, and TNF-α, and were significantly higher in the YM and OM groups than those in the YC and OC groups (**Figures 2A, B, E, H**). Furthermore, serum levels of pro-inflammatory cytokines MCP-1, CD40L, KCIL8 and IL-13 in the OM group were significantly higher than those in the OC group (**Figures 2G, I, K, N**). Serum levels of pro-inflammatory cytokines include GM-CSF, MCP-1, TNF-α, CD40L, KC and IL-13, and were significantly higher in the OM group than those in the YM group (**Figures 2A, G–I, K, N**), while the level of IL-10 was lower in the OM group compared to that in the YM group (**Figure 2M**). The OM and YM group did not differ in the levels of IFN-γ, IL-1β, IL-7, LIX, IL-17, and IL-12 (**Figures 2B, C, E, F, J, L**). The levels of GM-CSF, INF-γ, IL-7, LIX, TNF-α and IL-12 were significantly higher in the OC group than those in the YC group (**Figures 2A, B, E, F, H, L**), while the level of IL-10 was lower in the OC group compared to that in the YC group (**Figure 2M**). The OC and YC group did not differ in levels of IL-1β, IL-6, MCP-1, CD40L, IL-17, KC, and IL-13 (**Figures 2C, D, G, I–K, N**).

### Local Vascular Inflammation Analysis

Aging aggravated the plaque development and peripheral inflammatory response. CD68, a marker of the macrophage, stained more cells in the OM group than in the YM group (1.647 ± 0.429 *vs*. 1 ± 0.176, *P*<0.05; **Figures 1D, L**).

**Figure 3:**
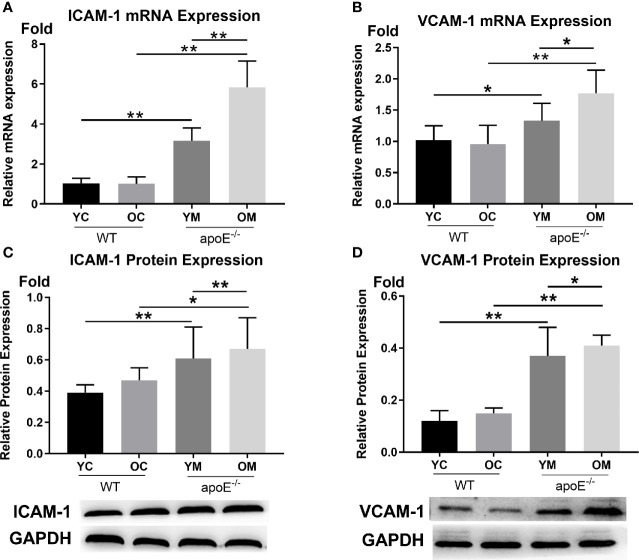
Transcription levels and expression levels of ICAM-1 and VCAM-1 in the aortas, n=6 mice per group. **(A, B)** Quantification of ICAM-1 and VCAM-1 transcription; **(C, D)** Quantification of ICAM-1 and VCAM-1 protein expressions. Data were shown as mean ± SD. ^*^ *p <*0.05; ^**^ *p <*0.01. *p* values are calculated from T-test.

In **Figures 3A, B**, the transcription levels of ICAM-1 (YM: 3.16 ± 0.64 *vs*. YC:1.03 ± 0.25, *P*<0.01; OM: 5.84 ± 1.31 *vs*. OC:1.01 ± 0.34, *P*<0.01) and VCAM-1 (YM: 1.33 ± 0.28 *vs*. YC:1.02 ± 0.23, *P*<0.05; OM: 1.77 ± 0.37 *vs*. OC:0.96 ± 0.30, *P*<0.01) in the entire aorta was both higher in the OM and YM groups than those in the YC and OC groups, respectively. Aging by itself caused a significant increase of ICAM-1 (*P*<0.01) and VCAM-1 (*P*<0.05) when comparing the YM groups with the OM groups, while no difference was detected between the YC and OC groups.

In **Figures 3C, D**, the protein expression levels of ICAM-1 (YM:0.61 ± 0.20 *vs*. YC:0.39 ± 0.05, *P*<0.01; OM: 0.67 ± 0.20 *vs*. OC:0.47 ± 0.08, *P*<0.05) and VCAM-1 (YM:0.37 ± 0.11 *vs*. YC:0.12 ± 0.04, *P*<0.01; OM: 0.15 ± 0.02 *vs*. OC:0.12 ± 0.04, *P*<0.01) in the aorta were both significantly higher in the YM and OM groups than those in the YC and OC groups. What is more, ICAM-1 and VCAM-1were both higher in the OM group than those in the YM group (*P <*0.01*, P <*0.05), while no difference was detected between the YC and OC groups.

### Fecal Microbiota Profile

#### Distribution of Gut Microbes

**Figure 4:**
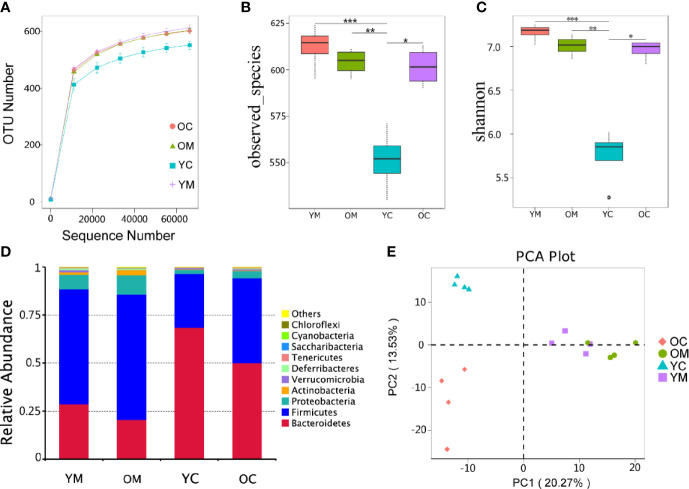
Effect of aging on microbiota composition, n=4 mice per group. **(A)** Rarefaction curves for OTU number in four groups. The curve in each group all reaches plateau; **(B, C)** α diversity (as accessed by observed species and shannon index) based on the general profile in the four groups (***p=0.0004, YM vs YC; *p = 0.0116, OC vs YC; for observed species; ***p=0.0001, YM vs YC; *p = 0.0115, OC vs YC; for Shannon; *p* values are calculated from Wilcoxon rank sum test); **(D)** Composition of fecal microbiota at the phylum level of four group; **(E)** PCA plot of microbiota communities cluster in feces of four group.

To characterize the bacterial richness, a rarefaction analysis was performed. The curves in all groups reach a plateau, which suggests that the size of the sequencing data was large enough, so few genes were ignored (**Figure 4A**). Alpha diversity, as confirmed by the number of Observed species and Shannon index, was much lower in the YC group compared to those in the YM, OM, and OC groups (*P*<0.01, *P*<0.01 and *P*<0.05; **Figures 4B, C**). Beta bacterial diversity was examined to measure the variation between samples. Principal Component Analysis (PCA) plots showed that the 16 samples were clustered into four distinct groups (PC1 axis:20.27% and PC2 axis:13.53%; **Figure 4E**). Analysis of Similarities (ANOSIM) also confirmed this clustering of the samples (YC *vs*. YM: *R*=1, *P*=0.036; OC *vs*. OM: *R*=1, *P*=0.028; YC *vs*. OC: *R*=1, *P*=0.033; YM *vs*. OM: *R*=0.5, *P*=0.024) (**Supplementary Figure S2**).

#### Intestinal Flora Composition

The species with the top 10 highest abundance on the phylum level of each group were selected to generate a columnar summation diagram of species relative abundance (**Figure 4D**). The dominating bacterial phyla (more than 95% of the sequences) consists mainly of *Bacteroidetes*, *Firmicutes*, and *Proteobacteria*. *Firmicutes* to *Bacteroidetes* ratio (3.17 *vs*. 2.08) and the relative abundance of *Proteobacteria* (10.1% *vs*. 7.6%) were much higher in the OM group than those in the YM group (**Table S2**).

**Figure 5:**
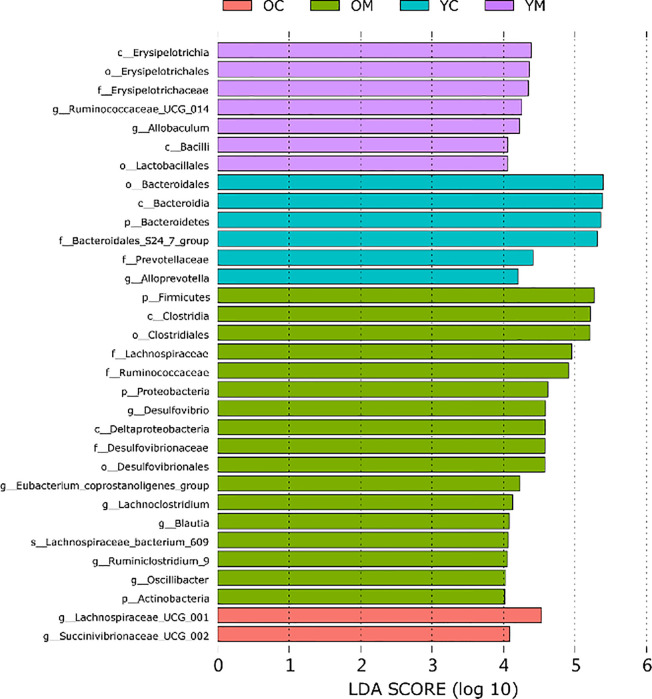
Differently enriched bacterial taxa among four groups which is determined using LEfSe algorithm, n=4 mice per group.

Furthermore, Linear discriminant analysis (LDA) coupled with effect size measurements (LEfSe) further identified dominant microbiota that accounted for the greatest differences observed among these groups (only taxa meeting LDA≥4 is shown, **Figure 5**). The abundances of genus *Oscillibacter*, genus *Eubacterium coprostanoligenes group*, genus *Ruminiclostridium_9*, genus *Desulfovibrio*, and phylum *Actinobacteria* were much higher in the OM group than those in the other three groups. Furthermore, the abundance of the family *Bacteroidales_S24_7_group* and the genus *Alloprevotella* were much lower in the OM, YM, and OC groups than that in the YC group. *Lachnospiraceae bacterium_609* and genus *Lachnoclostridium* were much more abundant in the OM and OC groups than those in the YM and YC groups, respectively.

#### Intestinal Microbial Metabolites

**Figure 6:**
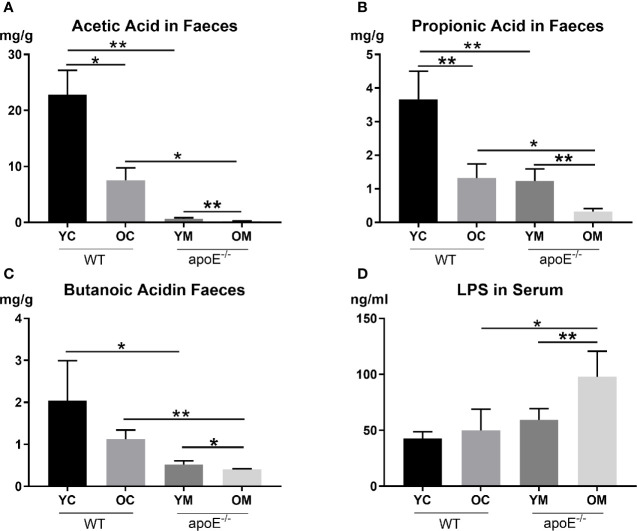
**(A–C)** Quantification of SCFAs levels in fecal, n=4 in each group; **(D)** Quantification of serum LPS level, n=4 in each group. Data were shown as mean ± SD. *p <0.05; **p <0.01. *p* values are calculated from T-test.

We next examined two core metabolites of gut microbes: short-chain fatty acids (SCFAs) and lipopolysaccharides (LPS). Compared with the age matched controls, the YM group demonstrated lower SCFA concentrations in the fecal sample than the YC group, such as Acetic Acid (0.67 ± 0.16 mg/g *vs*. 22.82 ± 4.35 mg/g, *P*<0.01; **Figure 6A**), Propionic Acid (1.23 ± 0.36 mg/g *vs*. 3.66 ± 0.84 mg/g, *P*<0.01; **Figure 6B**), and Butanoic Acid (0.52 ± 0.09 mg/g *vs*. 2.04 ± 0.95 mg/g, *P*<0.05; **Figure 6C**); However, serum LPS showed no significant difference between the YM and YC group. The OM group had lower serum Acetic Acid (0.23 ± 0.03 ng/ml *vs*. 7.52 ± 2.22 ng/ml, *P*<0.05; **Figure 6A**), Propionic Acid (0.32 ± 0.09 mg/g *vs*. 1.32 ± 0.42mg/g, *P*<0.05; **Figure 6B**), and Butanoic Acid (0.41 ± 0.01 mg/g *vs*. 1.12 ± 0.22, *P*<0.01; **Figure 6C**), but a higher serum LPS concentration (97.67 ± 23.07 ng/ml *vs*. 49.99 ± 18.95 ng/ml, *P*<0.05; **Figure 6D**) than those in the OC group.

Comparing the two control groups, the OC group displayed lower serum Acetic Acid and Propionic Acid levels in the fecal sample than those in the YC group (*P*<0.05, *P*<0.01 respectively; **Figures 6A, B**). On the other hand, serum SCFAs (Acetic Acid, Propionic Acid, and Butanoic Acid) in the OM group were all significantly lower compared to those in the YM group (*P*<0.01, *P*<0.01 and *P*<0.05; **Figures 6A–C**) while serum LPS concentration increased remarkably in the OM group compared to that in the YM group (*P*<0.01; **Figure 6D**).

#### Ileal Mucosal Barrier Function Analysis

**Figure 7:**
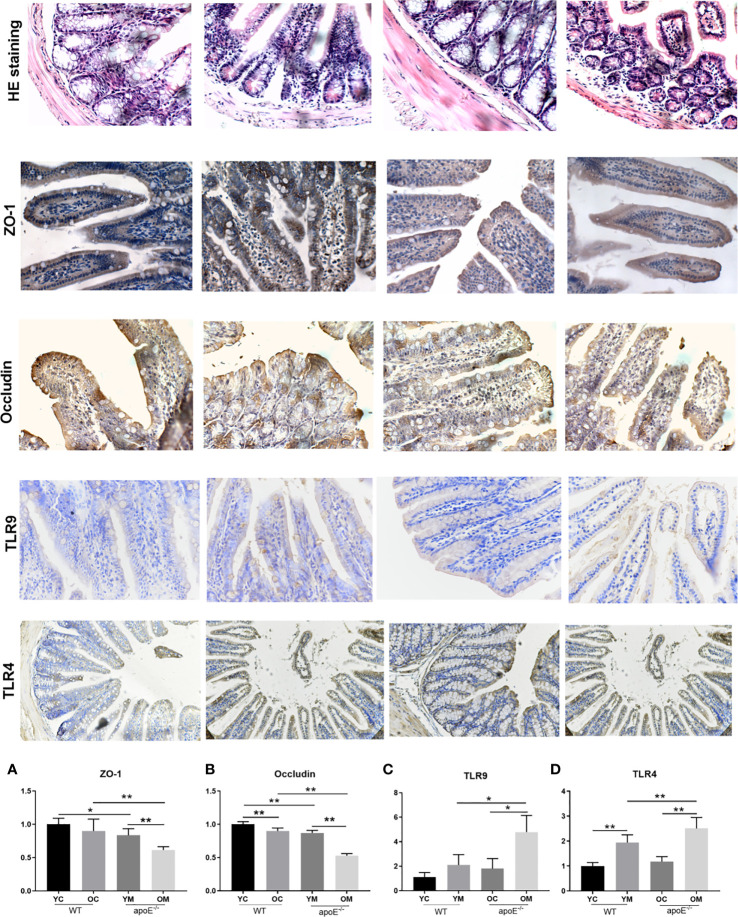
Quantification of ileal mucosal barrier dysfunction, n=4 in each group. HE staining and immunohistochemical staining of ZO-1, Occludin, TLR-9 and TLR-4 in ileal mucosal; The rest of the columns represent the expression levels of ZO-1, Occludin, TLR-9 and TLR-4 in ileal mucosal. Data were shown as mean ± SD. *P<0.05; **P < 0.01. p values are calculated from T-test.

As shown in HE staining of ileum, intact ileac villus structure with neatly arranged villus, mucosal epithelium integrity, and a few inflammatory cells infiltration in the mucosa were observed in the YC group. Mild abnormality in the villus was appreciated in the OC group. By contrast, the YM group showed deteriorating pathological alterations, such as scattered intestinal villi, an atrophied ileal mucosal layer, reduced mucosal thickness, and inflammatory cell infiltration in the mucosa. These changes were more severe in the OM group than in the YM group (**Figure 7**). The number of ZO-1 positive cells (YC *vs*. YM: *P*<0.05; OC *vs*. OM: *P*<0.01; **Figure 7A**) and OCLN positive cells (YC *vs*. YM: *P*<0.01; OC *vs*. OM: *P*<0.01; **Figure 7B**) in the gut was significantly decreased in the YM and OM groups than those in the age matched controls. Moreover, the number of OCLN positive cells was significantly decreased in the OC group than that in the YC group (*P*<0.01). Furthermore, the expressions of ZO-1 (*P*<0.01) and OCLN (*P*<0.01) in the aorta in the OM group was significantly decreased compared with that in the YM group. The expressions of TLR-4 and TLR-9 in the ileal mucosa in the YM and OM groups, were significantly higher than those in the age matched controls and their expressions were more obvious in the OM group than in the YM group (**Figures 7C, D**). Aging therefore deteriorated the ileal mucosal barrier disruption and enhanced endotoxin release in the blood stream, which is more obvious in the OM group than in the YM group.

#### Correlation Between the Gut Microbiome and Inflammatory Response

The correlation matrix between the gut bacterial and serum inflammatory cytokines, two core metabolites, SCFAs and LPS of gut the microbes’ changes was calculated by Pearson’s correlation coefficient (**Supplementary Figure S3**). There were significant correlations between the perturbed gut microbiome and the inflammatory response (*p < 0.05, **p <0.01). *Lachnospiraceae_FCS020_group* was significantly increased in the OM group, which was highly positively correlated with inflammatory cytokines ICAM-1, VCAM-1, KCIL8, CD40L, MCP-1, GM-CSF, and serum LPS; *Ruminococcaceae_UCG-009* was significantly increased in the OM group, which was highly positively correlated with inflammatory cytokines CD40L and MCP-1; *Acetatifactor* is positively associated with MCP-1 while *Lachnoclostridium* is positively associated with GM-CSF, which was also significantly increased in the OM group. Moreover, the OM group showed lower SCFA concentration in the fecal sample than that of the YM group, which is negatively associated with the genus of intestinal microbiota including *Lachnospiraceae_FCS020_group*, *Ruminococcaceae_UCG-009*, *Acetatifactor*, and *Lachnoclostridium*.

### Serum Metabolomic Analysis

#### Multivariate Statistical Analysis

**Figure 8:**
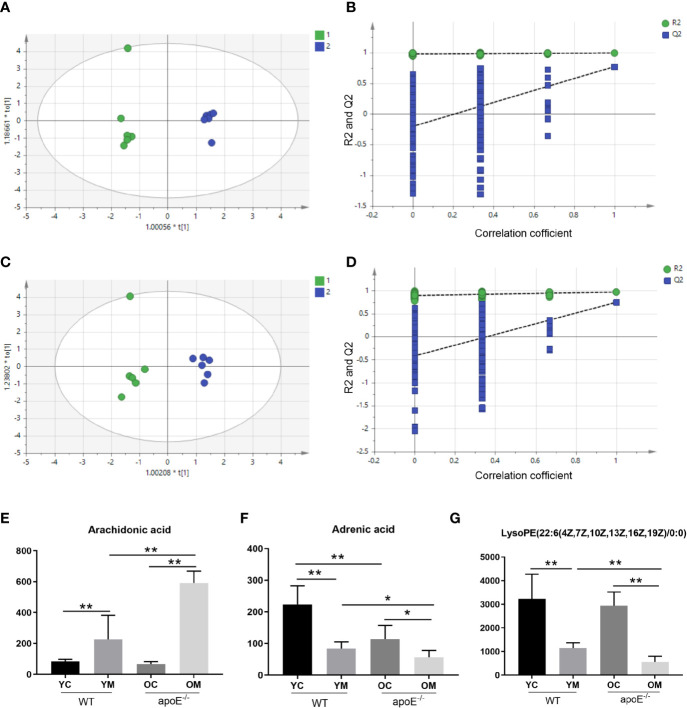
PLS-DA plots and validation plots for discriminating YM (1) and OM (2) in ESI+ and ESI- modes. **(A)** PLS-DA plot in ESI+ mode; **(B)** validation plot in ESI+ mode; **(C)** PLS-DA plot in ESI- mode; **(D)** validation plot in ESI- mode. Peak area of biomarkers in WT and apoE^-/-^ groups mice in positive ion and negative ion modes: **(E–G)**. PLS-DA, partial least squares discriminant analysis score; ESI+, electron spray ionization in positive; ESI-, electron spray ionization in negative; ESI, electron spray ionization; Q2, second quartile; R2, coefficient of determination; PLS-DA, partial least squares discriminant analysis score; horizontal axis t, principal component one; vertical axis t, principal component two. Data were shown as mean ± SD. ^*^ *p <*0.05; ^**^ *p <*0.01. *p* values are calculated from T-test, n=6.

We have performed positive (ESI+) and negative (ESI-) mode BPI chromatograms of serum samples obtained from all four groups by UPLC-Q/TOF-MS (**Supplementary Figures S4** and **S5**). Metabolic analysis revealed many metabolic differences between the YM group and OM group and their age matched controls. The OPLS-DA was used to identify differential metabolites between the YC group and YM group in the ESI+ mode (**Supplementary Figure S6A**) and ESI- mode (**Supplementary Figure S6B**), between the OC group and the OM group in the ESI+ mode (**Supplementary Figure S7A**) and ESI- mode (**Supplementary Figure S7B**), between the YM group and OM group in the ESI+ mode (**Figure 8A**) and ESI^-^ mode (**Figure 8B**). The PLS-DA models contained two predictive components of R2 and Q2 values. Comparing the YC group with the YM group in the ESI+ mode reveals R2X=0.602; R2Ycum=1; cumulative second quartile (Q2cum)=0.917, while R2X=0.711; R2Ycum=0.999; Q2cum=0.955 was shown in the ESI- mode. On the other hand, comparing the OC group with the OM group in the ESI+ mode revealed R2X=0.684; R2Ycum=1; cumulative second quartile (Q2cum)=0.95, while R2X=0.613; R2Ycum=0.998; Q2cum=0.953 were gained in the ESI- mode. The results between the YM and OM groups are shown in **Table S4**. The results suggest that the OPLS-DA model provides excellent fits and is highly predictive. The intercept values indicate that the model is stable and reliable without over fitting.

#### Screening and Identification of Metabolites Abundances

A two-class OPLS-DA model was constructed to observe the cluster patterns in each group. It was shown that the metabolic profiles of the YM and OM groups in the ESI+ and ESI- modes were clearly separated from that of the age matched controls. Following screening and the discovery of biomarkers, we then identified target metabolites as those with VIP > 1 and *p* < 0.05 in one-way ANOVA. The chemical structures of the metabolites were identified according to online databases such as the Human Metabolome Database (HMDB) and 63 metabolites of the YM and YC groups were obtained from the comparative analysis, including 35 metabolites in positive mode and 28 metabolites in negative mode. Furthermore, 55 metabolites of the OM and OC groups were obtained from the comparative analysis, including 27 metabolites in positive mode and 28 metabolites in negative mode.

Moreover, 27 metabolites, which served as early biomarkers of AS were obtained from the comparative analysis, including 11 metabolites in positive mode and 16 metabolites in negative mode (**Table S4**). Among these, the levels of trans-Aconitic acid, 3-Hydroxypicolinic acid, Leukotriene B4, 9,10-Epoxyoctadecenoic acid, Prostaglandin F2a, 15-HEPE, 20-Hydroxyeicosatetraenoic acid, Arachidonic acid, Docosapentaenoic acid (22n-6), Pyruvic acid, 15(S)-Hydroxyeicosatrienoic acid, Isocitric acid, Retinal, Tetradecanedioic acid, alpha-Dimorphecolic acid, Retinyl ester (*p*< 0.01), and L-Carnitine, Sphingosine 1-phosphate (*p* < 0.05) were higher in the OM group than those in the YM groups. And the levels of 3-Hydroxyisovalerylcarnitine, 1-Methylhistamine, 3-Hydroxypicolinic acid, LysoPE (22:6(4Z,7Z,10Z,13Z,16Z,19Z)/0:0) (*p* < 0.01) and Indolelactic acid, Adrenic acid, DL-2-Aminooctanoic acid, 12(13) Ep-9-KODE, and LysoPC (14:0/0:0) (*p* < 0.05) were lower in the OM group than those in the YM groups. All these indicate a correlation with aging (**Supplementary Figure S8**; A-Z_0_). Among these differential metabolites, three metabolites demonstrate a significant difference between the OM group with the YM groups, as well as between the YM group and OM group with the YC group and OC group, respectively. They are Arachidonic acid (**Figure 8E**) and LysoPE (22:6(4Z,7Z,10Z,13Z,16Z,19Z)/0:0) in the ESI+ mode (**Figure 8F**) and Adrenic acid in the ESI- mode (**Figure 8G**).

### Correlation Between Gut Microbiome and Metabolites in apoE^-/-^ Groups

The correlation matrix between the metabolites and gut bacterial changes was generated by calculating the Pearson’s correlation coefficient (**Supplementary Figure S9**). There were clear correlations between perturbed gut microbiome and altered metabolite profiles (*p < 0.05, **p <0.01). The metabolite 1-methylhistamine was significantly decreased in the OM group, which is highly associated with the genus of intestinal microbiota, such as *Ruminococcaceae_UCG-014*, *Akkermansia*, and *Parasutterella*. Among the microflora mainly appearing in the OM group, *Lachnospiraceae_FCS020_group* was obviously positively correlated with trans-Aconitic acid, isocitric acid, 20-hydroxyeicosatetraenoic acid (20-HETE), and Tetradecanedioic acid. Moreover, *Ruminococcaceae_UCG-009* is associated with serum metabolites such as L-Carnitine, Prostaglandin F2a (PGF2α), Arachidonic acid, and Leukotriene B4 (LTB4).

### Analysis of Metabolic Pathways

The 27 early biomarkers of AS were imported into the MetPA database to analyze the related pathways. It generated six networks (**Supplementary Figure S10**) including arachidonic acid metabolism, retinol metabolism, pyruvate metabolism, histidine metabolism, sphingolipid metabolism, and biosynthesis of unsaturated fatty acids (**Table S5**). The pathway impact value was calculated from a pathway topology analysis, and the threshold was set as Raw *P* < 0.01 and impact < 0.1. The unique pathway was filtered out as the potential pathway (Arachidonic acid metabolism) to AS. The results suggested that such a pathway showed a marked impact over the advanced stage in AS which could contribute to the development of AS. Metabolic pathways of the KEGG database were linked through the differentially abundant metabolites, as shown in **Supplementary Figure S10B**.

## Discussion

The increase in life expectancy will lead to an increased prevalence of age-related cardiovascular diseases. AS is the pathological basis of cardiovascular disease, so its underlying molecular mechanisms is worth attention. Clinical studies showed that aged (65-85 years) people developed more complex aortic plaques than middle-aged (45-64 years) people (Kong et al., 2019). Therefore, the role of aging in AS is key to understanding the underlying metabolic changes during atherosclerotic evolution.

ApoE, a multifunctional protein, is expressed by many cell types, and affects many aspects of cardiovascular physiology, such as macrophage cholesterol homeostasis and systemic lipid metabolism. ApoE deficient (apoE^-/-^) mice can spontaneously develop atherosclerosis which stimulate atherosclerotic pathological processes in the arteries of humans with lesions that can be observed during the development of atherosclerosis (Nakashima et al., 1994). Furthermore, aged apoE^-/-^ mice (20 to 40 weeks of age) developed more advanced, fibrous plaques, with abundant fibrous tissue and larger necrotic cores (Jakic et al., 2019). To observe the effect of aging on more severe atherosclerosis lesions, we compared the plaque areas in young (5-week) and old (32-week) apoE^-/-^ mice with the intervention of a high fat diet for 32 weeks, another risk factor for AS, to ensure the formation of complex plaques (Yu et al., 2018). HE staining of the aortic root showed that apoE^-/-^ mice developed complex atherosclerotic lesions with the cholesterol crystals and calcium deposition. Compared to the OM group, the YM group presented a milder change in the lesion composition. Assessments of morphologic changes of the entire aorta and aortic root demonstrated that aged mice were more susceptible to complex aortic atheroma plaques than the young mice.

It is generally accepted that the formation of the AS plaque is highly dependent on dyslipidemia, which triggers the accumulation of lipids in the intimal artery walls followed by lipid core formation, innate immune cell recruitment, and activation (Lacy et al., 2019). We therefore assessed the serum lipid levels in these mice. Both young and aged ApoE^-/-^ mice suffered from dyslipidemia upon HFD intervention as expected. Interestingly, a comparison of the YM group with the OM group revealed that the aged mice exhibited a notable increase in TC levels but decreased HDL and TG levels, and similar LDL levels. Oil red O staining showed that there was no difference in lipid accumulation within the plaque between these two groups. Taken together, we found that dyslipidemia in these mice failed to explain the reason why the aged ApoE^-/-^ mice suffered more severe AS lesions than the young mice. Further exploration of the underlying mechanisms of these phenomena is needed.

Moreover, our work showed that the levels of serum proinflammatory cytokines GM-CSF, MCP-1, TNF-α, CD40L, KC, and IL-13 were significantly increased while the anti-inflammatory cytokine IL-10 was significantly decreased in aged mice than in young mice, as shown in **Figure 2**. The aged mice were therefore in a state of chronic low-grade inflammation known as inflammaging (Calder et al., 2017) which is consistent with previous findings (Pedersen and Bruunsgaard, 2003; Sikora et al., 2014). All of these pro-inflammatory cytokines in turn lead to the activation of endothelial cell adhesion molecules (ECAMs), such as VCAM-1 and ICAM-1, which were reported to regulate monocytes/macrophages recruitment (Pang et al., 2019). Macrophages are well known as innate immune cells and are a key player of AS because it mediates lipoprotein internalization and cytokine production that leads to localized inflammation and plaque growth (Zhou et al., 2015). We further demonstrated the elevation of gene transcription and protein expression of VCAM-1 and ICAM-1 in aortas, as well as the accumulation of macrophages in aged AS mice compared with their young counterparts. Briefly, inflammaging in aging might contribute to the development and progression of AS. So, the next question is: Where does the inflammaging arise from.

AS is a chronic inflammatory disease. In addition, recent studies have found that gut microbe disturbance is a major cause of AS. Accumulating evidence indicates that gut flora disturbance is closely related to inflammaging and the subsequent development of AS (Fandriks, 2017; Vaiserman et al., 2017; Herrema et al., 2020). The human body hosts a vast number of microorganisms in the gut. The interaction between these microbes and the host is complicated. Several microbes have been exhibited to affect the development of AS through the direct invasion of endothelial cells, followed by macrophage infiltration and vascular inflammation (Koren et al., 2011). Additionally, gut bacterial release LPS and peptidoglycan into circulation which act as strong inducers of a systemic inflammatory response *via* ECAMs, as described above (Shi et al., 2014). Furthermore, severely disturbed gut flora and decreased SCFAs production could induce a systemic inflammatory response *via* the activation of the intestinal immune system (Winer et al., 2016).

We then used 16S rDNA sequencing to evaluate the composition of the intestinal flora and the distribution of specific microbes. A HFD for 8-20 weeks combined with aging was shown to greatly reduce gut flora diversity in mice (Thacker et al., 2016). By contrast, our study showed that the diversity of the gut flora had no significant difference between the YM and OM groups in **Figures 4B, C**. The prolonged diet intervention of 32 weeks may contribute to these phenomena. However, the beta diversity analysis showed that the intestinal microbiota of mice in the two different age groups presented different discrete aggregation. Furthermore, previous reports indicated that an increased ratio of Firmicutes to Bacteroides was related to a HFD (Tomas et al., 2016). We found that the ratio of Firmicutes to Bacteroides and the relative abundance of Proteobacteria increased in aged ApoE^-/-^ mice consuming a HFD than in their younger counterparts, as shown in **Table S2**. Interestingly, there was a significant decrease in the quantities of beneficial microbes in the families *Ruminococcaceae-UCG-014* and an increase in *Lachnospiraceae_FCS020*, *Ruminococcaceae_UCG-009*, *Acetatifactor*, *Lachnoclostridium*, and *Lactobacillus gasseri* in the feces of aged mice than in the feces of their younger counterparts. A HFD can induce an imbalance of the intestinal flora, especially an increase in the abundance of endotoxin-producing *Desulfovibrio* which damages the gut barrier function and results in high levels of circulating LPS (Zhang et al., 2010; Zhang et al., 2012). In our study, aged apoE^-/-^ mice had a significantly higher relative abundance of *Desulfovibrio* compared with the other three groups. Moreover, the genus beneficial *Bacteriodes*, a group of short-chain fatty acid (SCFA)-producing bacteria (Yan et al., 2020) was markedly decreased in HFD-fed apoE^-/-^ mice. The analysis proved a significantly altered composition in intestinal flora in old AS mice compared to their younger counterparts. In summary, an aging-induced structural imbalance of intestinal flora is closely related to the development of AS in this study.

Imbalance of the intestinal flora can lead to increased permeability of the intestinal wall and dysfunction of the intestinal mucosal barrier (Zeng et al., 2019). In **Figure 7**, lower expression of tight junction protein ZO-1 and occludin accompanied with the intestinal mucosal barrier disorder were observed in the aged group which might explain why their serum LPS concentrations were remarkably increased. Moreover, inflammation in the ileal mucosal with TLR-4 and TLR-9 accumulation were shown in the aged group in our study. Toll-like receptors (TLRs) are key sensors for recognition of these bacterial molecules (Akira and Hemmi, 2003). TLR4 is activated by LPS to induce pro-inflammatory mediators. Moreover, TLR9 plays a key role in protecting the integrity of the intestinal epithelium and defending against S. typhimurium infection (Li et al., 2017). This study confirmed that TLR4 and TLR-9 modulate the inflammatory response. Furthermore, SCFAs are produced by fermentation of dietary fibers by the intestinal flora, which play a vital role in maintaining the intestinal mucosal barrier integrity and in the inflammatory response (Smith et al., 2013; Chen et al., 2017). In accordance with the modifications of structure and composition in gut flora, the SCFAs in the fecal sample also decreased with aging. It was reported that decreased SCFAs correlate with gut barrier dysfunction and with the epithelium inflammatory response (Li et al., 2018; Hu et al., 2019). In turn, it increases the permeability of the gut barrier to endotoxin molecules such as LPS, therefore the translocation of SCFA from gut lumen into circulation was allowed. Furthermore, association analysis on intestinal microbes and serum LPS, inflammatory cytokines showed that the decreased intestinal microbiota such as *Ruminococcaceae-UCG-014* is negatively correlated with the production of inflammatory factors and LPS in serum, while the increased intestinal microbiota *Lachnospiraceae_FCS020*, *Ruminococcaceae_UCG-009*, *Acetatifactor*, *Lachnoclostridium*, and *Lactobacillus_gasseri* were positively correlated with its production. All those intestinal microbiota were negatively correlated with acetate, propionate and butyrate. These SCFAs can strongly stimulate the activation of human monocytes and reduce proinflammatory cytokine and chemokine production by monocytes (Nastasi et al., 2015). Some genera of *Ruminococcaceae* produce acetate and butyrate, not only serving as the main source of energy of intestinal epithelial cells but also inhibiting the signaling pathway of proinflammatory cytokines (Morgan et al., 2012).

The serum metabolome results indicated that arachidonic acid (AA) metabolism elevated markedly in aged AS mice compared with their younger counterparts. AA metabolites through different pathways to Arachidonic acid, LTB4, PGF2α, and 20-HETE which were significantly increased in the serum of aged AS mice versus their younger counterparts. Arachidonic acid is a major polyunsaturated fatty acid, from which some inflammatory mediators are derived in mammals (Yagami et al., 2018). Arachidonic acid can be metabolized by lipoxygenases (LOX) (Lehmann et al., 2015) to leukotrienes (LT), or by cyclooxygenases (COX) (Willenberg et al., 2015) to prostaglandins (PGs) and thromboxanes (TXs), or by cytochrome P450s (CYP450) and LOX (Fleming, 2011) to hydroxyeicosatetraenoic acids (HETEs) and hydroxyoctadecadienoic acids (HODEs) (Wolfer et al., 2017). Development and progression of AS is a complicated process regulated by many internal factors, including arachidonic acid-derived lipid mediators. It was shown that AA and its derivatives link nutrient metabolism to immunity and inflammation, thus playing a key role in the initiation and progression of AS (Demetz et al., 2014). In our study, Arachidonic acid, LTB4, 20-HETE and PGF2α were of higher levels in the OM groups than in the YM groups. These results indicate that aging causes arachidonic acid metabolic dysfunction and exacerbates the progression of AS.

Moreover, more than 30% of metabolites in the human body come from gut microbes that may contribute to host diseases (Holmes et al., 2012). An association analysis on intestinal microbes and metabolites suggested, for the first time, a close positive correlation between *Lachnospiraceae_FCS020_group* and 20-HETE, between *Ruminococcaceae_UCG_009* and Prostaglandin F2a, Arachidonic acid, and Leukotriene B4. On the other hand, those microbiomes enriched in young mice including *Bacteroidia* and *Prevotellaceae* exhibit an evident negative relationship with AA metabolites of 20-HETE. *Lachnospiraceae_FCS020_group*, as an IL-6 positive-related genus, induces an inflammatory response in the body (Tang et al., 2018). A significantly decreased relative abundance of *Ruminococcaceae_UCG_009* in the colon is of an anti-inflammatory effect (Zha et al., 2019). Moreover, *Bacteroidetes*, a gram-negative bacillary obligatory anaerobe, plays an important role in maintaining immune homeostasis and regulating immunity, which is associated with unsaturated fatty acids (Wang et al., 2020). Finally, we speculate that alterations in gut microbes and metabolites may contribute to exacerbated inflammatory responses and dysregulated immune function in aged AS mice.

In this study, we found that aged AS mice showed more severe AS lesions and a pronounced inflammatory response than their younger counterparts. We then used 16S rRNA sequencing and metabolomics profiling to study the impact of aging on the gut microbes and metabolic profiles. The data clearly showed that aging induced a significant change in the gut microbiome composition among WT and apoE^-/-^ mice. Moreover, these perturbed gut microbes were strongly associated with differential alternation of metabolites between the YM and OM groups. It indicates that aging not only disturbs gut microbes but also substantially alters host metabolite homeostasis. Ultimately, we found that arachidonic acid metabolism was the main metabolic pathways involved in the inflammatory response of aging. These findings may provide new insights of how to delay AS development in aged patients.

## Conclusions

Aging not only alters the gut microbiome community but also substantially disturbs metabolic conditions. Our results confirm that AA metabolism is associated with intestinal flora disturbance in aging AS lesions. These findings may provide novel insights regarding how gut flora disorders and gut microflora–related metabolites induce inflammaging. Potential new mechanisms of how aging leads to or exacerbates AS could be further explored based on this study.

## Data Availability Statement

The original contributions presented in the study are publicly available. This data can be found here: NCBI PRJNA682554.

## Ethics Statement

The animal study was reviewed and approved by Animal Management Committee of Tianjin University of Traditional Chinese Medicine (会(批准号 TCM LAEC2019039). Written informed consent was obtained from the owners for the participation of their animals in this study.

## Author Contributions

YS drafted the manuscript. XJ designed and supervised the experiments as the PI. BL, QG, YC, DW, WZ, LY, and HL critically revised the manuscript. All authors reviewed and approved the final manuscript.

## Funding

This work was supported by the National Natural Science Foundation of China [NO. 82074211, NO. 81804025], Tianjin Municipal Natural Science Foundation [NO. 18JCYBJC94500], Scientific and Technological Research Program of Tianjin Municipal Education Commission [NO.2017KJ164], and Research and Innovation Project of Graduate [YJSKC-20191003].

## Conflict of Interest

The authors declare that the research was conducted in the absence of any commercial or financial relationships that could be construed as a potential conflict of interest.
