# Supplementary material for: The Role of Intestinal Dysbacteriosis Induced Arachidonic Acid Metabolism Disorder in Inflammaging in Atherosclerosis
Source: Front Cell Infect Microbiol. 2021 Mar 18;11:618265. doi: 10.3389/fcimb.2021.618265 (PMC8012722; doi:10.3389/fcimb.2021.618265)
Supplement: Supplementary file 1 [file DataSheet_1.pdf]

## Supplementary Materials

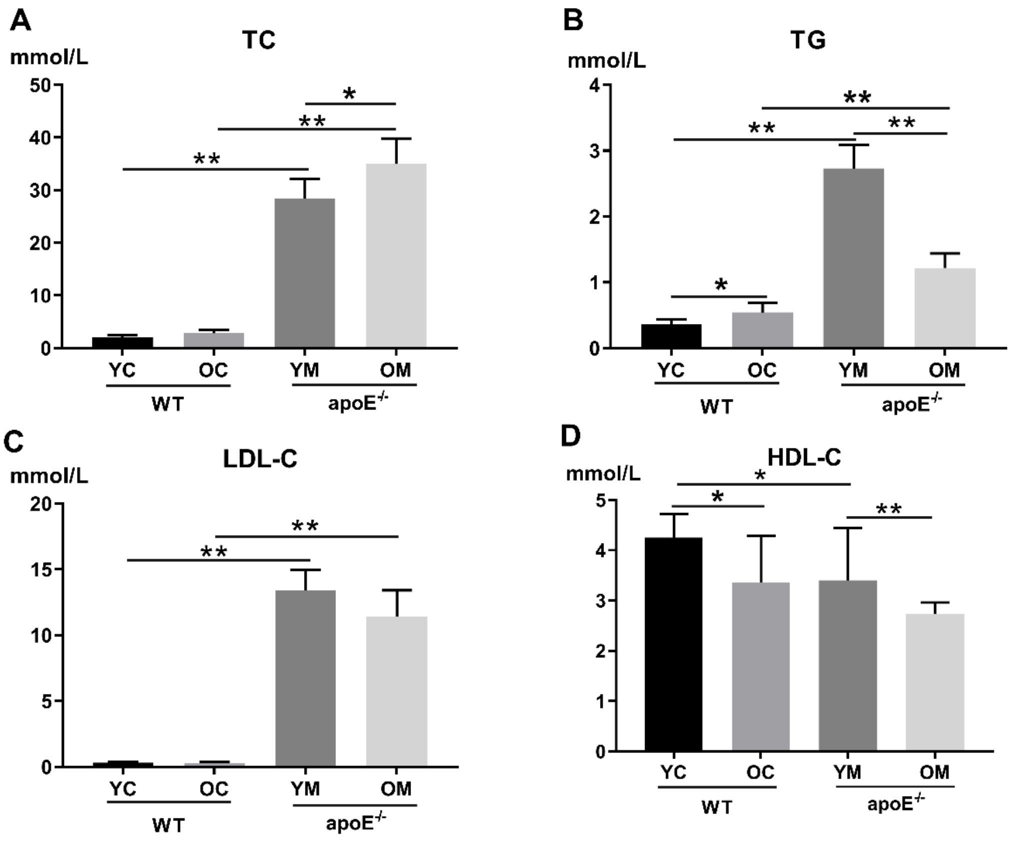


**Fig. S1**: Quantification of serum lipid profile, n=6 each group. (A) TC; (B) TG; (C) LDL-C; (D) HDL-C. Data were shown as mean ± SD. $^{*}$ *p* <0.05; $^{**}$ *p* <0.01. *p* values are calculated from T-test.

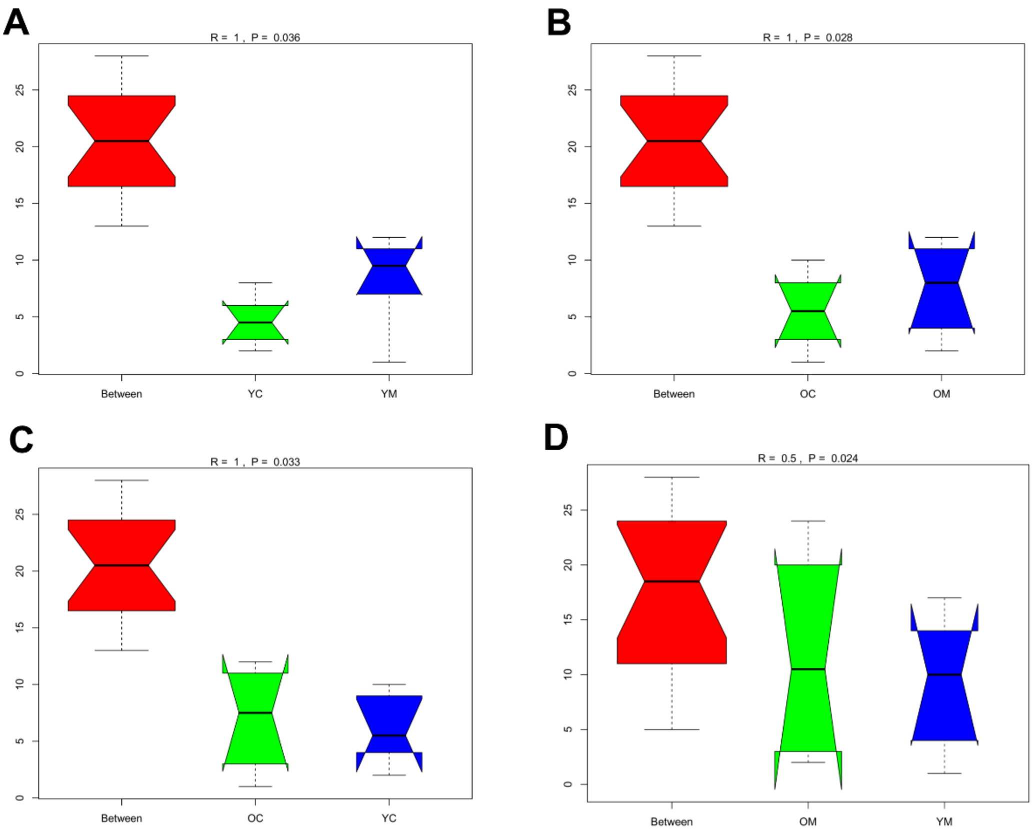


**Fig. S2**: The ANOSIM which generates an R test statistic stretching from −1 to 1 was used to measure clustering of samples. A positive R value indicates greater within-group similarity than between-group similarity, Greater R value suggests stronger clustering of samples. An R value of 0 indicates no clustering of samples, yet a negative R value suggests greater between-group resemblance than within-group similarity.

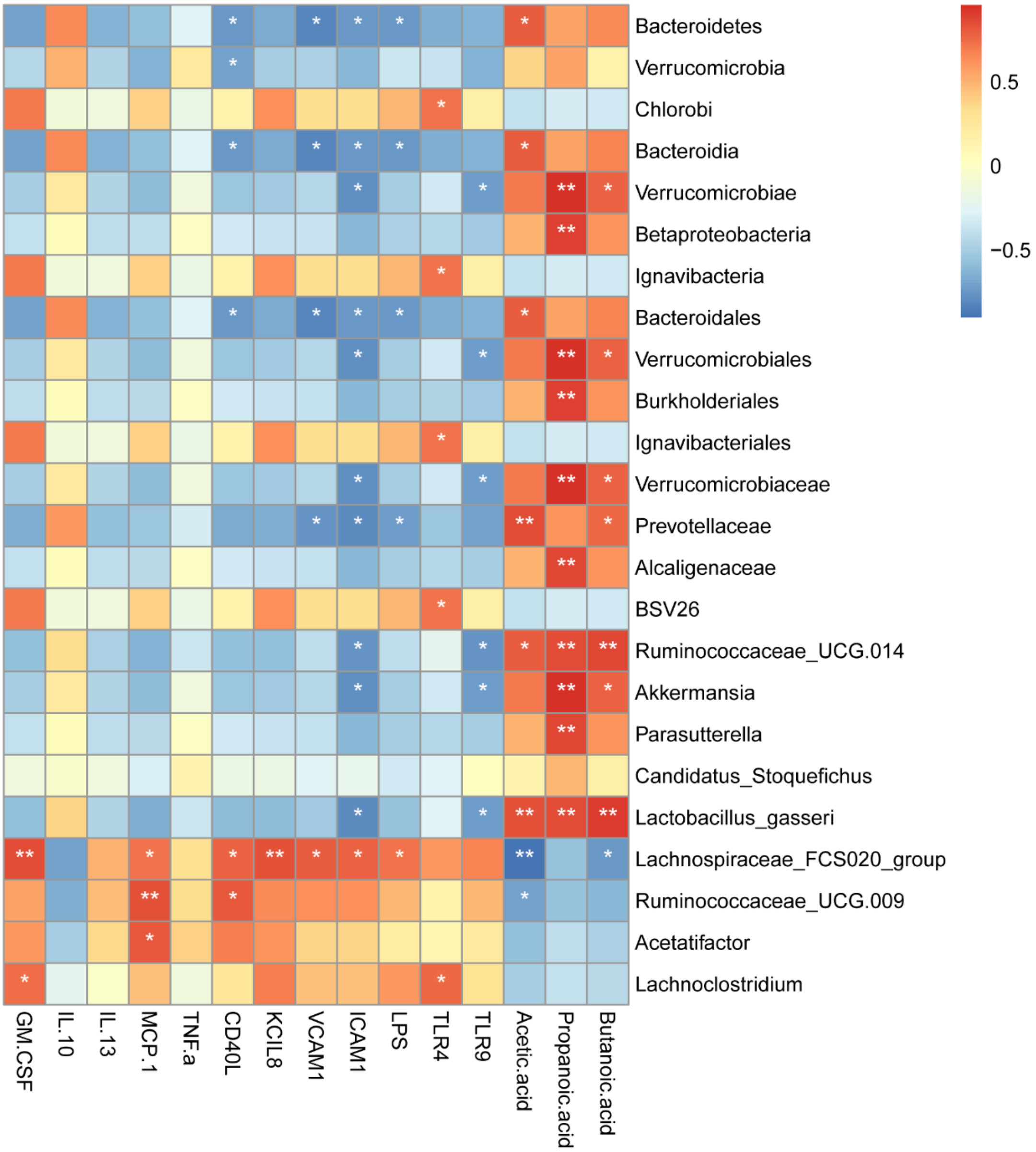


**Fig. S3**: Correlation analysis between specific microflora wich serum levels of LPS and inflammatory cytokines, with faecal levels of SCFAs, TLR4 and TLR9,with inflammatory cytokines ICAM-1, VCAM-1 in AS plaques. *p <0.05; **p <0.01. p values are calculated by Pearson Correlation Coefficient, n=4.

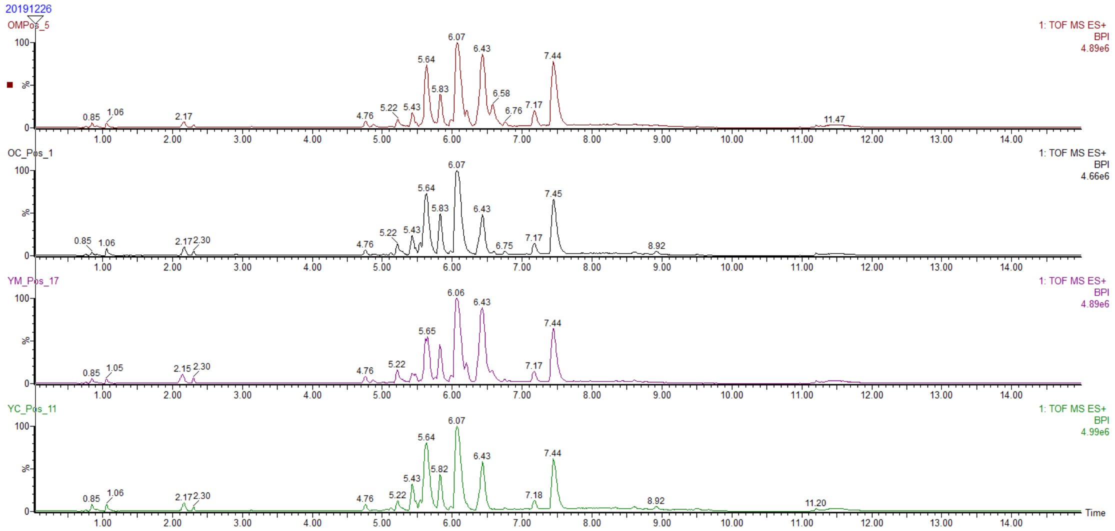


**Fig. S4.** Comparison of positive ion mode analysis to serum samples from four groups.

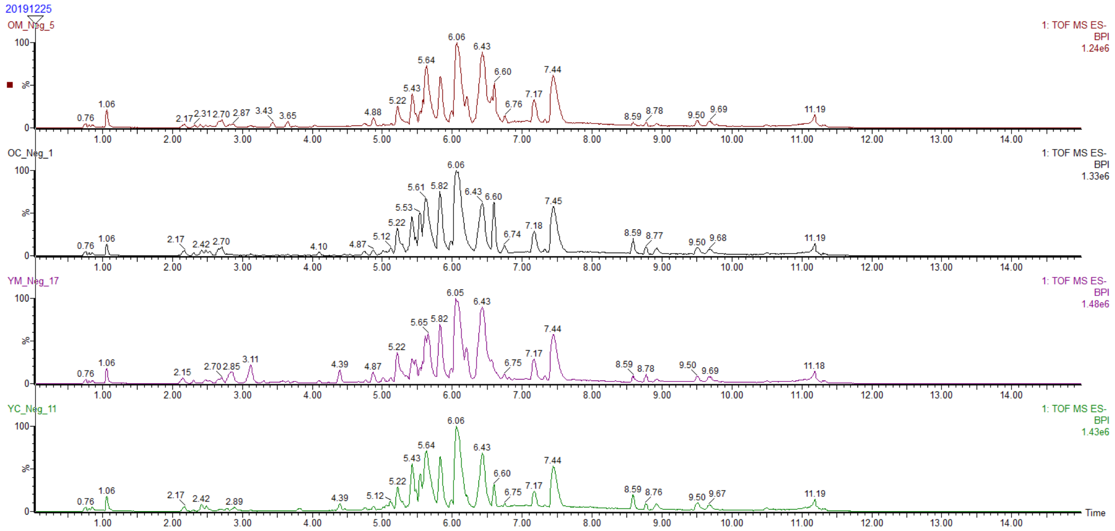


**Fig. S5.** Comparison of negative ion mode analysis to serum samples from four groups.

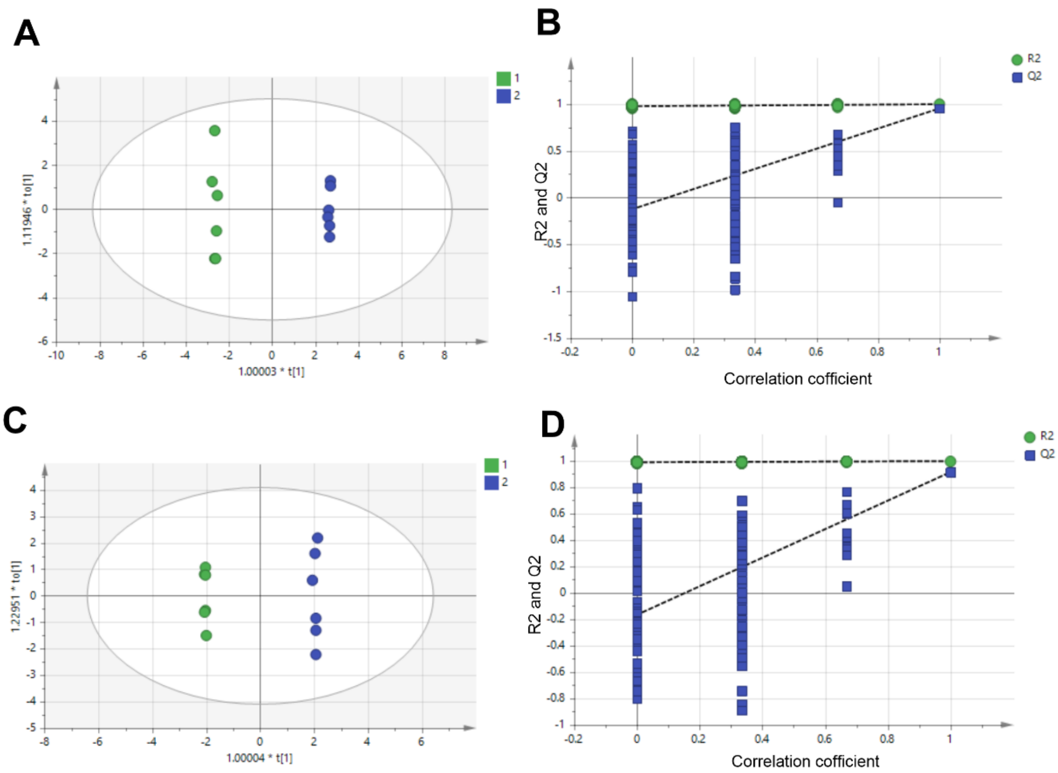


**Fig. S6.** PLS-DA plots and validation plots for discriminating YC (1) and YM (2) in ESI+ and ESI- modes. (A) PLS-DA plot in ESI+ mode; (B) validation plot in ESI+ mode; (C) PLS-DA plot in ESI- mode; (D) validation plot in ESI- mode.

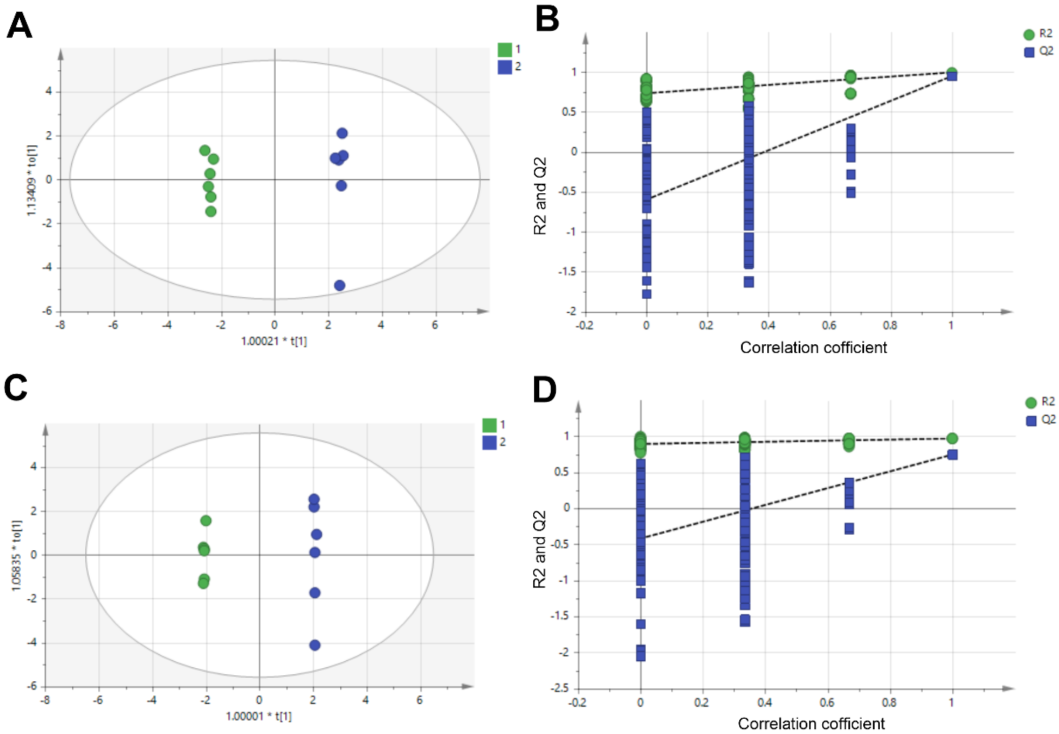


**Fig. S7.** PLS-DA plots and validation plots for discriminating OC (1) and OM (2) in

ESI+ and ESI- modes. (A) PLS-DA plot in ESI+ mode; (B) validation plot in ESI+ mode; (C) PLS-DA plot in ESI- mode; (D) validation plot in ESI- mode.

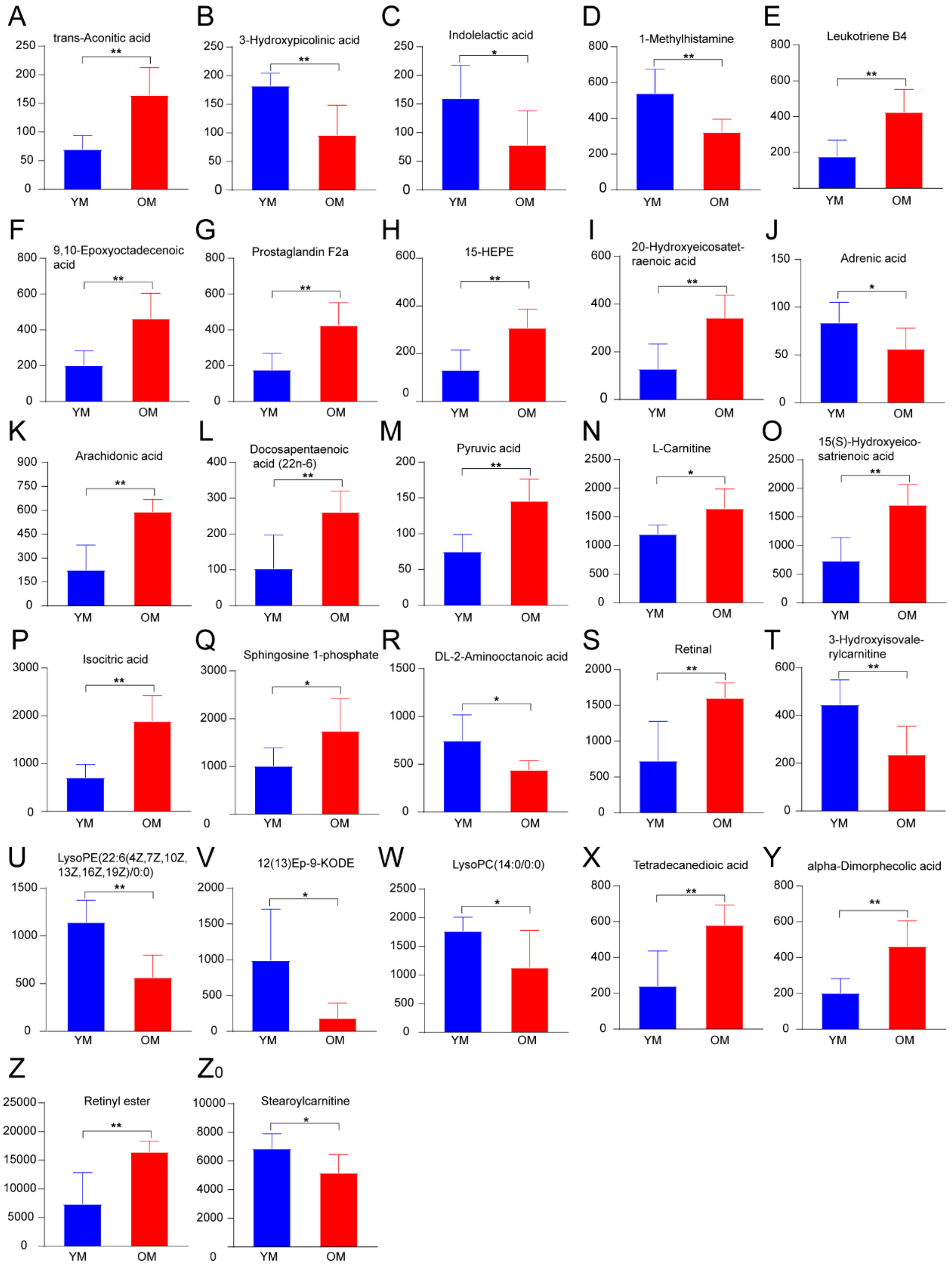


**Fig. S8.**: Peak area of AS biomarkers in YM and OM groups in positive ion and negative ion modes: (A-$Z_0$). Data were shown as mean ± SD. $^{*}p$ <0.05; $^{**}p$ <0.01. $p$ values are calculated from T-test, n=6.

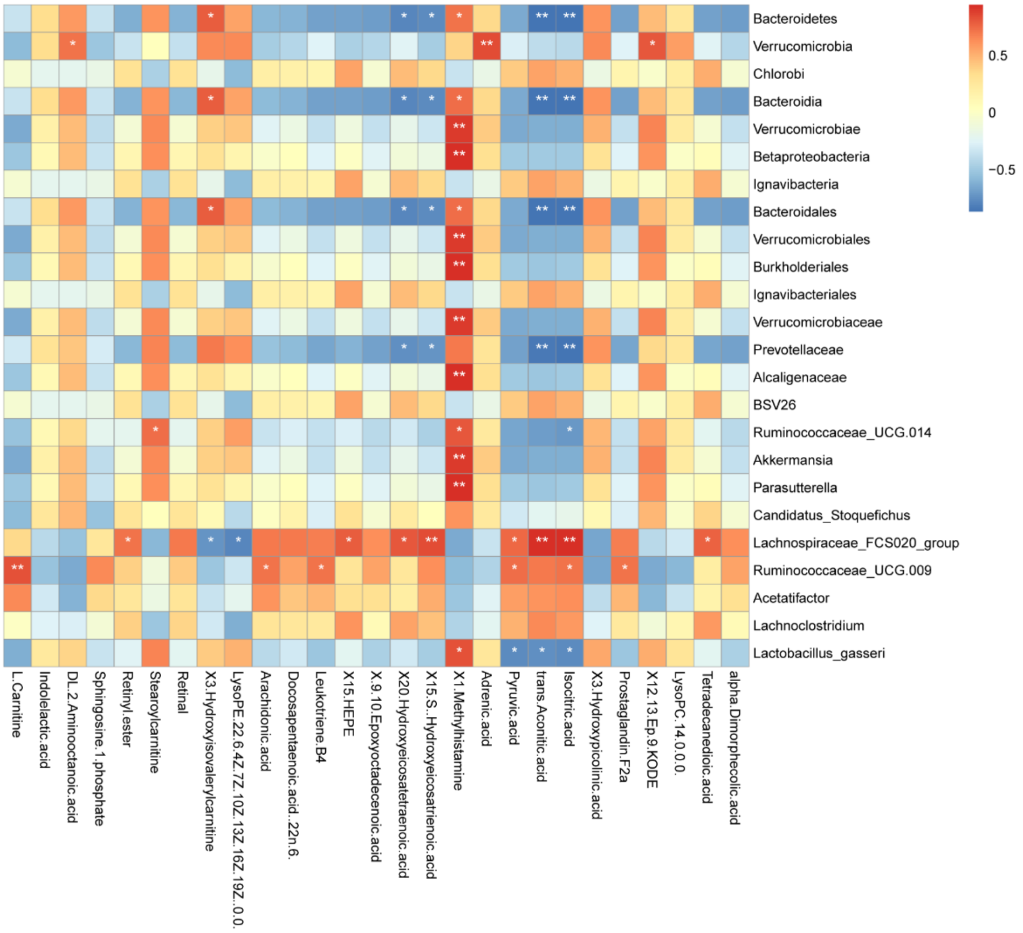


**Fig. S9.** Correlation analysis between the specific microflora with serum differential metabolites. Data were shown as mean ± SD. $^{*}p$ <0.05; $^{**}p$ <0.01. $p$ values are calculated by Pearson Correlation Coefficient, n=4.

A

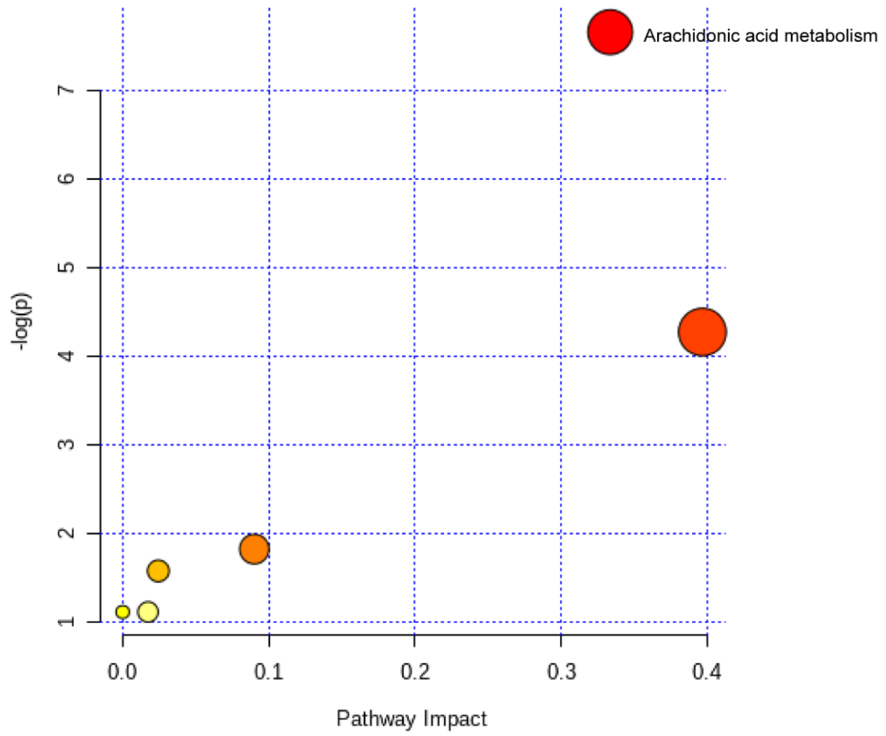


B

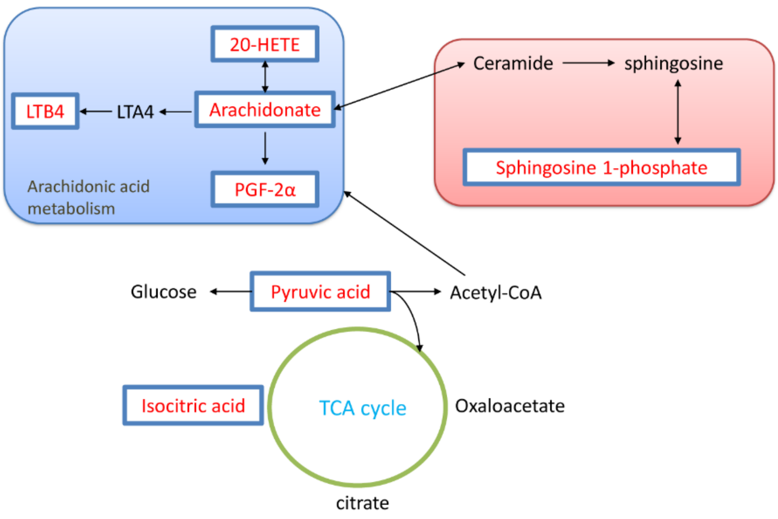


**Fig. S10.** Summary of pathway analysis and network analysis. (Red labeled metabolites: potential biomarkers with increased levels in OM group, compared to that in YM group.)
